# Risk factors related to pleural empyema after talc slurry pleurodesis

**DOI:** 10.1016/j.clinsp.2022.100098

**Published:** 2022-08-27

**Authors:** Paula Duarte D'Ambrosio, Pedro Henrique Xavier Nabuco de Araújo, Eserval Rocha Junior, Mauro Razuk Filho, Paulo Manuel Pêgo-Fernandes, Ricardo Mingarini Terra

**Affiliations:** a Instituto do Câncer do Estado de São Paulo (ICESP), Hospital das Clínicas HCFMUSP, Faculdade de Medicina, Universidade de São Paulo, São Paulo, SP, Brazil; b Instituto do Coração, Hospital das Clinicas HCFMUSP, Faculdade de Medicina, Universidade de São Paulo, São Paulo, SP, Brazil

**Keywords:** Empyema, Pleurodesis, Pleural Effusion, Malignancy

## Abstract

**Objective:**

Empyema is a complication of talc-pleurodesis that may lead to further surgical intervention and death. Therefore, the present study's objective was to identify the risk factors for the development of post-pleurodesis empyema after talc slurry pleurodesis in order to better select patients for this procedure and minimize its morbidity.

**Methods:**

Patients with malignant pleural effusion who underwent talc slurry pleurodesis at the present institution from January 2018 to January 2020 were retrospectively analyzed. Post-pleurodesis empyema was defined as pleural infection up to 30 days after pleurodesis. Using Cox regression analysis, significant prognostic factors for the development of empyema were examined.

**Results:**

Of the 86 patients identified for inclusion in the study, 62 were women (72%). Their mean age was 56.3±12.6 years. The median pleural drainage time was 9 days, and 20 patients (23.3%) developed empyema. In the univariate analysis, both drainage time (*p* = 0.038) and the use of antibiotics prior to pleurodesis (*p* < 0.001) were risk factors for pleural empyema. Multivariate analysis also identified the use of antibiotics as an independent risk factor (Odds Ratio [OR] 9.81; 95% Confidence Interval [95% CI] 2.87‒33.54). Although the pulmonary expansion was not associated with empyema in the multivariate analysis, patients with less than 50% pulmonary expansion had a 4.5-times increased risk of empyema (95% CI 0.90‒22.86; *p* = 0.067), and patients with 50‒70% pulmonary expansion had a 3.8-times increased risk of empyema (95% CI 0.98‒15; *p* = 0.053) after pleurodesis.

**Conclusion:**

The study suggests that antibiotic therapy prior to talc slurry pleurodesis may increase the risk of developing empyema. Furthermore, pleurodesis should be considered with caution in patients with long-duration chest tube placement and incomplete lung expansion.

## Highlights

• The study's objective was to identify the risk factors for the development of post-pleurodesis empyema in order to better select patients for this procedure and minimize its morbidity. The study suggests that antibiotic therapy prior to pleurodesis may increase the risk of developing empyema. Furthermore, pleurodesis should be carefully considered in patients with long-duration chest tube and incomplete lung expansion.

## Introduction

Malignant Pleural Effusion (MPE) is an evident sign of advanced cancer and a disabling condition that will occur in 50% of patients with metastatic cancer. In 2012, up to 126825 hospital admissions in the United States were secondary to MPE.^1^ The management of patients with MPE is palliative, and pleurodesis is the most common method used to alleviate symptoms and prevent pleural effusion recurrence.^2^

Pleurodesis is an accepted therapy to provide effective control of recurrent MPE in patients with disseminated pleural neoplastic disease.^3^ However, despite proven efficacy, talc pleurodesis has also been associated with potentially serious systemic and local complications. Although the incidence of empyema after talc pleurodesis is low, the rate of morbidity and mortality from this complication is high because the patients affected are often fragile and weakened by advanced cancer.^3^^,^^4^ According to de Campos and colleagues, the incidence of empyema after talc poudrage can be as high as 4.0%.^4^ Other studies suggest that the incidence of empyema may be rare after talc slurry pleurodesis, however.^3^

Unfortunately, few series have specifically analyzed the hazard of empyema after talc slurry pleurodesis; therefore, the risk factors for empyema post-talc slurry are still unknown. Due to the clinical relevance of this major complication in a frail patient population, the present study's aim was to identify the risk factors associated with empyema in patients with MPE undergoing talc slurry pleurodesis.

## Materials and methods

This was a retrospective study performed at the Instituto do Câncer do Estado de São Paulo (ICESP), Hospital das Clínicas HCFMUSP, Faculdade de Medicina, Universidade de São Paulo. The study was approved by the Institutional Review Board (CAPPesq HCFMUSP ‒ 4612195).

**Fig. 1:**
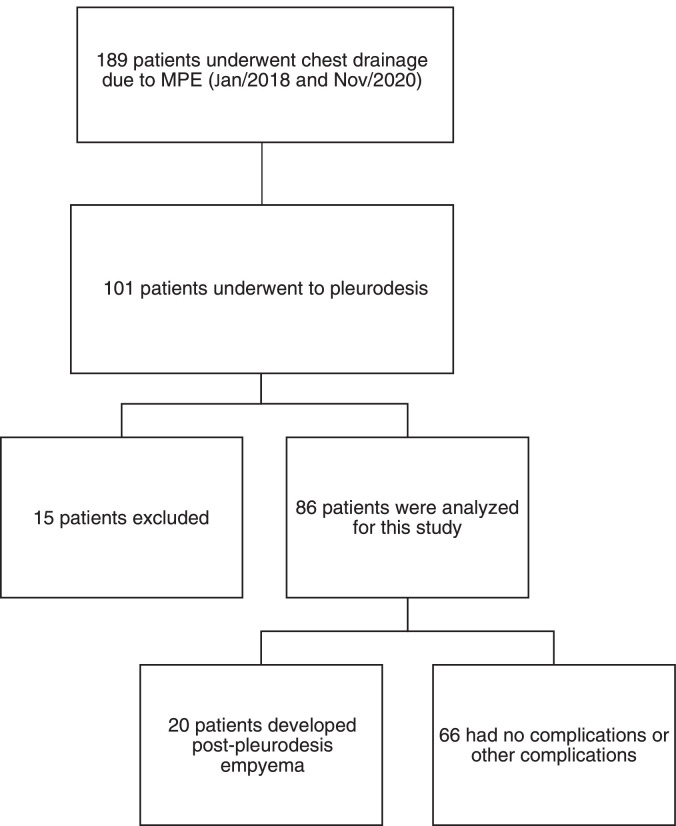
Flowchart.

The authors retrospectively reviewed the electronic medical records for the present chest drainage database of 189 patients who had undergone chest drainage for malignant pleural effusion between January 2018 and November 2020. Only 101 patients were identified as having pleurodesis performed using talc slurry. Patients younger than 18 years of age or with current pleural infection, previous major pleural procedures (pleurodesis or decortication), hemorrhagic diathesis, pleurodesis with another agent, neoplastic skin infiltration at the drainage site, benign etiology, or incomplete data were excluded from the study (n = 15) (Fig. 1). All talc slurry pleurodesis procedures were performed by board-certified thoracic surgeons.

Usually, after a few days of pleural drainage, talc slurry pleurodesis was carried out in a procedure room located in the emergency department or in an outpatient clinic. In all patients, the skin was prepped with alcoholic chlorhexidine, and the surgical field was isolated with sterile drapes. After local anesthetic (lidocaine 2.0%) administration, pleural drainage was carried out with a 14 Fr pigtail catheter (C-UPTP-1400-WAYNE, Cook Medical, Bloomington, IN) using the trocar technique. A Heimlich valve was connected to the drain, and its distal end was connected to a collection bag. Using a sterile technique, talc slurry pleurodesis was performed using 3.0g of non-graded talc (Magnesita Refratarios SA: Contagem, Brazil) administered through the pigtail catheter in 60 mL of saline solution and 5.0 mL lidocaine 2.0%). The solution was injected, and the tube was clamped for 1–2 hours. The removal of the pleural catheter was based on fluid volume and the patient's clinical condition. Systemic analgesia was administered only when necessary.

Basic demographic information including age, sex, type of malignancy, comorbidities, previous treatment received (previous surgery, chemotherapy, and radiotherapy), initial performance status via the Eastern Cooperative Oncology Group (ECOG) index, and data regarding the pleurodesis procedure were extracted from electronic medical records. Pleural catheters were placed by the Thoracic Surgery Service, and the anatomical location of placement varied according to each patient's necessity. Patients were considered eligible for pleurodesis if they had recurrent MPE with diagnosis confirmed by oncotic cytology of pleural fluid or pleural biopsy and Karnofsky Performance Score (KPS) > 30.

**Fig. 2:**
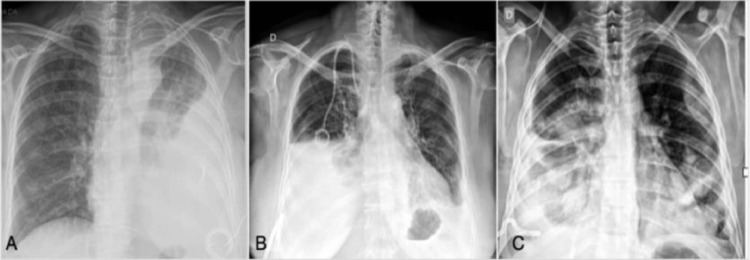
Classification of chest radiographs according to lung pleural expansion after drainage. Caption: (A) Lung pleural expansion less than 50% on the left side, (B) lung pleural expansion between 50‒70% on the right side, (C) Lung expansion more than 70% on the right side.

Chest radiographs were retrieved and graded according to the percentage of pleural cavity that was occupied by the lung after the drainage (less than 50%, between 50% and 70%, and more than 70%) (Fig. 2). The classification was performed independently by two thoracic surgeons. When an agreement between them was not achieved, the senior author was consulted and asked to make a determination.

**Fig. 3:**
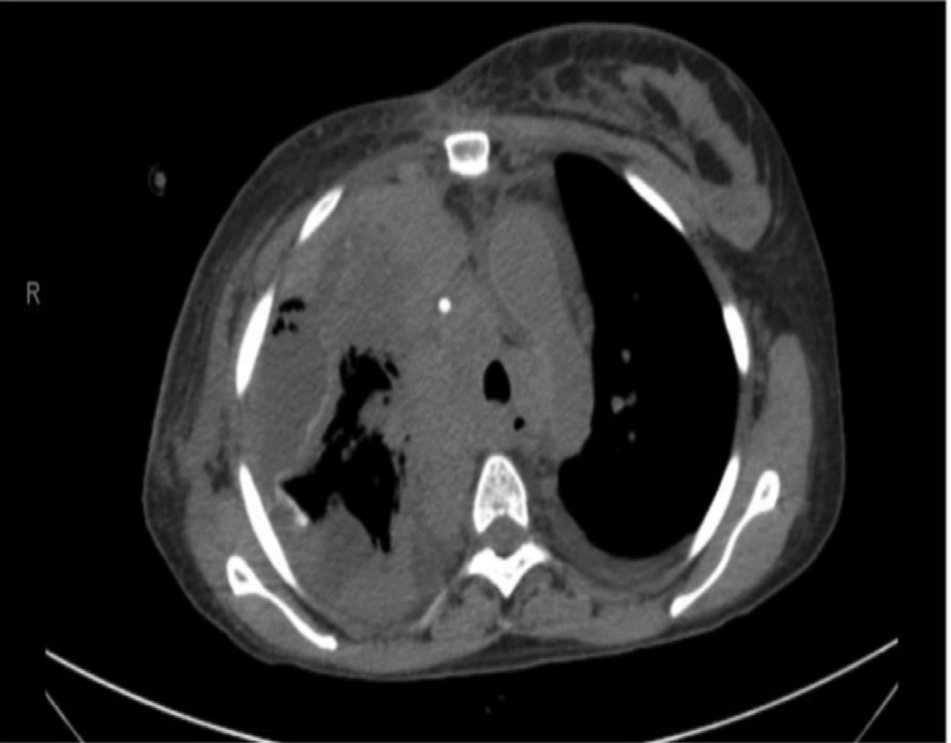
Empyema post-pleurodesis on chest CT scan. Caption: Chest CT scan (axial) after talc pleurodesis, showing a right malignant pleural effusion loculated, pleural calcification secondary to talc, pleural thickening, and intervening gas suggestive of empyema.

Post-pleurodesis empyema was defined as the development of pleural infection up to 30 days after the procedure, according to Centers for Disease Control and Prevention: Atlanta, Georgia, United States criteria (CDC).^5^ The diagnosis of post pleurodesis empyema was based on an analysis of the pleural effusion according to the American Association for Thoracic Surgery (AATS) guidelines^6^ with consideration of symptoms, laboratory investigations, and radiological exams. Patients with a clinical picture highly suggestive of pleural empyema but who had not undergone any pleural fluid analysis were independently evaluated by a multidisciplinary team that included thoracic surgeons and radiologists, and the diagnosis of empyema was established by consensus. The empyema, an adverse event, was graded according to the Common Terminology Criteria for Adverse Events (CTCAE) v.5.0 instrument,^7^ as exemplified in (Fig. 3).

### Statistical analysis

Descriptive statistical analysis was used to summarize the characteristics of the studied patients and surgical procedures. Frequencies and percentages are presented for categorical variables, and continuous variables are summarized as the median. Multiple variable models using logistic regression. Variables with p < 0.05 in univariable analysis were retained in the final model. All statistical analyses were performed using the software SPSS (IBM Corp) version 20.0. Probability (p) values of less than 0.05 were considered statically significant.

## Results

Of the 86 patients who underwent talc slurry pleurodesis and were included in the study, 24 were men (28%) and 62 were women (72%); their median age was 56.3 years (range 29‒83) (Table 1A). The median pleural drainage time before talc slurry was 8 days (range 6.0‒9.0). Twenty-six patients (30.2%) received antibiotic therapy in the 15 days before pleurodesis; 35 (40.7%) received antibiotics during the 15 days after pleurodesis. Radiological examinations showed that 12 patients (14%) had lung expansion after thoracic drainage that was less than 50%, 28 (32.5%) had 50‒70% expansion, and 46 (53.5%) had greater than 70% lung expansion (Table 1B).

After talc slurry pleurodesis, 20 patients (23.3%) developed post-pleurodesis empyema (Table 1B). Eleven patients had grade 4 post-pleurodesis empyema, 7 patients had grade 3 empyema, and 2 patients had grade 1 empyema as assessed using the CTCAE grading system. Thirteen patients (65%) that developed empyema received antibiotical treatment before being subjected to talc-pleurodesis. Among the 20 patients who developed empyema, 7 patients underwent a surgical procedure (debridement in 4, decortication in 2, and thoracostomy in 1). Six patients were referred to palliative care and were offered comfort measures with no further interventions.

In univariate analysis, the use of antibiotics before pleurodesis and drainage time prior to pleurodesis were risk factors for the development of empyema (Table 2). Treatment with antibiotics before pleurodesis was associated with a 7.57 increased risk of empyema as compared with no antibiotic treatment (Odds Ratio [OR] = 7.57; 95% Confidence Interval [95% CI] 2.52‒22.77; p < 0.001) (Table 2). Moreover, each additional day of drainage before pleurodesis increased the risk of empyema a factor of 1.11 (OR = 1.11; 95% CI 1.01‒4.53; *p* = 0.038) (Table 2). No other clinical variables had a significant association with the development of empyema in the univariate analysis.

**Table 1A:** Characteristic of study group.

Variables		
Sex	Female	62 (72.1)
	Male	24 (27.9)
Age, n (Max‒Min)		56.3 (29**‒**83)
Comorbidities, *n* (%)	Yes	42 (48.8)
	No	44 (51.2)
Etiology, *n* (%)	Breast	35 (40.7)
	Lung	21 (24.4)
	Others^a^	30 (34.9)
ECOG previous drainage, *n* (%)	0‒1	38 (44.0)
	2	27 (31.4)
	3‒4	21 (24.4)
Side of pleural effusion, *n* (%)	Right	56 (65.1)
	Left	29 (33.7)
	Bilateral	1 (1.2)
Previous use of antibiotics, *n* (%)	Yes	26 (30.2)
	No	60 (69.8)
Lung pleural expansion after drainage, *n* (%)	< 50%	12 (14)
	50‒70%	28 (32.5)
	> 70%	46 (53.5)
Drainage time before pleurodesis, *n* (Max‒Min)	Median	8 (6‒9)
Post-pleurodesis empyema, *n* (%)	Yes	20 (23.3)
	No	66 (76.7)

*n* = 86 patients.

a Esophagus, stomach, colon, rectum, biliary tract, pancreas, cervix, ovary, prostate, kidney, bladder, thyroid, multiple myeloma and myeloid leukemia.

**Table 1B:** Characteristic of study group.

Variables, *n* (%)		
**Post-pleurodesis empyema**	**Yes,***n* (%**)**	**No,***n* (%**)**
Total, n	20	66
**Previous use of antibiotics**		
Yes	13 (65)	13 (20)
No	7 (35)	53 (80)
**Drainage time before pleurodesis**		
Median	9	8
**Lung pleural expansion after drainage**		
< 50%	5 (25)	7 (11)
50‒70%	8 (40)	20 (30)
> 70%	7 (35)	39 (59)

*n* = 86 patients.

**Table 2:** Univariate model logistic regression analysis to identify factors associated with empyema post-pleurodesis.

Independent variables	p-value^a^	Odds Ratio (95% CI)^b^
Previous use of antibiotics	< 0.001	7.57 (2.52‒22.77)
Drainage time before pleurodesis (days)	0.038	1.11 (1.01‒1.23)
Lung expansion after drainage:		
< 50%	0.053	3.98 (0.98‒16.16)
50‒70%	0.172	2.23 (0.71‒7.03)
> 70%	1	

*n* = 86 patients.

a Significance probability.

b Confidence Interval 95%.

In the multivariate analysis, only the previous use of antibiotics remained a significant risk factor (OR = 9.81; 95% CI 2.87‒33.54; p < 0.001) (Table 3). The degree of pulmonary expansion after drainage was not significantly associated with the development of empyema in the multivariate analysis. Patients with < 50% lung expansion presented a 4.53 higher risk of empyema after pleurodesis (95% CI 0.90‒22.86; *p* = 0.067), and patients with pulmonary expansion between 50‒70%, as assessed by post-drainage X-Ray, had a 3.8 higher risk of empyema after pleurodesis (95% CI 0.98‒15.00; *p* = 0.053) (Table 3).

**Table 3:** Multivariate model logistic regression analysis to identify factors associated with empyema post-pleurodesis.

Independent variables	*p*-value^a^	Odds Ratio (95% CI)^b^
Previous use of antibiotics	< 0.001	9.81 (2.87‒33.54)
Lung expansion after drainage:		
< 50%	0.067	4.53 (0.90‒22.86)
50‒70%	0.053	3.84 (0.98‒15.00)

*n* = 86 patients.

a Significance probability.

b Confidence Interval 95%.

## Discussion

This study aimed to identify the risk factors associated with empyema after talc slurry pleurodesis. The univariate analysis showed that antibiotic treatment and prolonged drainage time prior to pleurodesis were risk factors for post-pleurodesis empyema. Patients who used antibiotics prior to the procedure were 7.57 more likely to have empyema than patients who did not. Even though the reduced lung expansion was not significantly associated with the risk of developing empyema, 65% of the patients who developed empyema had lung expansion inferior to 70%. In contrast, 35% of patients with lung expansion classified as greater than 70% in the radiographic analysis developed empyema after talc pleurodesis. In the multivariate analysis, the use of antibiotics prior to the procedure remained a significant risk factor for the development of empyema.

Talc pleurodesis, through a standard chest drain or via thoracoscopy, has been reported to yield high success rates for treating MPE.^8^ On the other hand, however, concerns over severe acute complications after talc administration have decreased the enthusiasm about its use, especially in patients with advanced malignancy.^9^ Despite the low incidence, empyema is considered a serious complication after talc pleurodesis, especially in patients with an average survival of fewer than 6 weeks and multiple medical co-morbidities.^10^ Moreover, the low incidence of post-pleurodesis empyema may reflect the difficulty in diagnosing this complication. The development of an infection in patients with MPE decreases their likelihood of survival and increases the patient's suffering, which may switch the care priorities to noninvasive and comfort measures. In this context, diagnosing empyema after a palliative procedure followed by an inflammatory response can be a challenge for the physician.

The present study reported a very high incidence of post-pleurodesis empyema as compared with an earlier study (23.3% vs. 4.0%).^3^ This might be due to the fact that the authors identified patients with empyema through a retrospective analysis of clinical data from the medical records. It is important to highlight those 6 patients did not have pleural fluid analysis and were considered to have post-pleurodesis empyema due to evidence found in the clinical data gathered retrospectively. It is possible that some patients in the present series, as well as in other series, were not diagnosed with empyema. The number of patients with empyema is likely underestimated after pleurodesis to treat MPE because clinicians may consider the worsening symptoms as the progression of end-stage disease in some patients. The increased incidence of empyema may also reflect the fact that the authors performed pleurodesis in patients who are not typically treated with pleurodesis at all centers, such as patients with < 70% lung expansion. This is because indwelling pleural catheters are not available in the Brazilian public health system. Furthermore, at the present study's institution, thoracentesis is an option only for patients with very short survival expectancy, low-performance status, and pleural lung expansion < 50%. The performance of thoracentesis is rarely the optimal approach for palliating dyspnea; relief of symptoms is usually short-lived, and repeated procedures are required.

Although empyema is associated with a mortality of 20% in the general population,^11^ few reports in the literature attempt to describe the risk factors for post-pleurodesis empyema. Earlier studies reported that 50% of the patients who developed post-pleurodesis empyema received antibiotics to treat non-pleural infections, and a multivariate regression analysis identified this factor as statistically significant. However, it is challenging to compare the present results with the results of others. In 2018, a mere 337 articles were published on “pleural empyema” as compared with more than 3000 articles on other complications with similar prevalence, rendering this condition “orphan” and in need of further investigation.^12^

From a logical perspective, full lung pleural expansion after drainage should be essential for a good outcome after pleurodesis.^13^ Surprisingly, however, one series with 87 patients with recurrent MPE and large pleural space, measured by CT scans immediately before bedside pleurodesis and 30 days after) identified in almost 40% of those patients, found successful pleurodesis in 86.2%, and 4.6% post-pleurodesis major complications were identified: one case of acute respiratory distress, one case of empyema, one case of pulmonary thromboembolism, and one case of sepsis.^14^ The authors observed that pleurodesis might be effective even in those patients without full lung expansion after drainage, and should not be contraindicated, even more, when IPC is not available[16].

In contrast, we found that more patients with < 50% lung expansion developed empyema as an adverse effect than patients with > 70% lung expansion (∼40% vs. 15%). Similarly, Terra and colleagues reported a high empyema rate (28.5%) in patients with trapped lungs and long-term drainage, although their study was limited by a small number of patients.^15^

The findings of this study allowed us to make informed clinical changes in the authors’ daily practice before performing talc pleurodesis. The procedure should be performed as early as possible to avoid long-duration drainage. Radiologic findings should be carefully assessed, and the talc-slurry pleurodesis should be avoided in patients who received antibiotics prior to the procedure.

The present study has several limitations, most inherent to the methodology. The main limitation of this study is its retrospective nature, which may have introduced selection biases. The single-center dataset is a similar limitation. With a larger sample size, some of the trends the authors observed may have become statistically significant. The determination of the primary outcome, empyema, based on medical record data may have overestimated its occurrence, but to our knowledge, the present study is the first to focus on post-pleurodesis empyema as the primary outcome. Prospective data collection would reduce these limitations, but the low incidence of empyema after talc pleurodesis would make this a lengthy undertaking.

## Conclusions

The results of this study suggest that treatment with antibiotics prior to talc slurry pleurodesis is a risk factor for developing empyema in patients with MPE. Additionally, in patients with a long-permanence chest tube or with incomplete lung expansion, the indications for pleurodesis should be carefully considered due to a higher risk of pleural empyema. Multi-center studies with a larger population are needed to better characterize the risk factors for pleural empyema in patients with MPE who undergo talc slurry pleurodesis.

## Authors’ contributions

Paula Duarte D'Ambrosio and Pedro Henrique Xavier Nabuco de Araújo conceived of the presented idea (conceptualization). Paula Duarte D'Ambrosio and Mauro Razuk Filho performed the measurements (methodology), processed the experimental data (data curation), performed the formal analysis, drafted the manuscript, and designed the figures. Pedro Henrique Xavier Nabuco de Araújo, Eserval Rocha Junior, Paulo Manuel Pêgo-Fernandes, Ricardo Mingarini Terra contributed to the interpretation of the results. Paula Duarte D'Ambrosio wrote the manuscript (original draft) with support from Mauro Razuk Filho. Paulo Manuel Pêgo-Fernandes and Ricardo Mingarini Terra supervised the project (project administration). All authors provided critical feedback

## Conflicts of interest

The authors declare no conflicts of interest.
